# Assessing Physician’s Motivational Communication Skills: 5-Step Mixed Methods Development Study of the Motivational Communication Competency Assessment Test

**DOI:** 10.2196/31489

**Published:** 2022-06-24

**Authors:** Vincent Gosselin Boucher, Simon Bacon, Brigitte Voisard, Anda I Dragomir, Claudia Gemme, Florent Larue, Sara Labbé, Geneviève Szczepanik, Kimberly Corace, Tavis Campbell, Michael Vallis, Gary Garber, Codie Rouleau, Jean G Diodati, Doreen Rabi, Serge Sultan, Kim Lavoie, Network For Health Behavior Change And Promotion (CAN-Change)

**Affiliations:** 1 Montreal Behavioural Medicine Centre Centre Intégré Universitaire de santé et services sociaux du Nord-de-l’Ile-de-Montréal (CIUSSSNIM) Montreal, QC Canada; 2 Department of Psychology Université du Québec à Montréal Montréal, QC Canada; 3 Department of Health, Kinesiology and Applied Physiology Concordia University Montreal, QC Canada; 4 Faculty of Medicine of Montpellier Montpellier France; 5 Department of Psychiatry University of Ottawa Ottawa, ON Canada; 6 The Royal’s Institute of Mental Health Research Ottawa, ON Canada; 7 Total Cardiology Cardiac Rehabilitation Calgary, AB Canada; 8 Department of Psychology University of Calgary Calgary, AB Canada; 9 Department of Family Medicine Dalhousie University Halifax, NS Canada; 10 Department of Medicine University of Ottawa Ottawa, ON Canada; 11 Department of Medicine University of Toronto Toronto, ON Canada; 12 Department of Community Health Sciences University of Calgary Calgary, AB Canada; 13 Department of Pediatrics Université de Montréal Montreal, QC Canada; 14 Canadian Network for Health Behavior Change and Promotion (CAN-Change) Montreal, QC Canada

**Keywords:** assessment, motivational communication, tool development, physicians, health promotion

## Abstract

**Background:**

Training physicians to provide effective behavior change counseling using approaches such as motivational communication (MC) is an important aspect of noncommunicable chronic disease prevention and management. However, existing evaluation tools for MC skills are complex, invasive, time consuming, and impractical for use within the medical context.

**Objective:**

The objective of this study is to develop and validate a short web-based tool for evaluating health care provider (HCP) skills in MC—the Motivational Communication Competency Assessment Test (MC-CAT).

**Methods:**

Between 2016 and 2021, starting with a set of 11 previously identified core MC competencies and using a 5-step, mixed methods, integrated knowledge translation approach, the MC-CAT was created by developing a series of 4 base cases and a scoring scheme, validating the base cases and scoring scheme with international experts, creating 3 alternative versions of the 4 base cases (to create a bank of 16 cases, 4 of each type of *base* case) and translating the cases into French, integrating the cases into the web-based MC-CAT platform, and conducting initial internal validity assessments with university health students.

**Results:**

The MC-CAT assesses MC competency in 20 minutes by presenting HCPs with 4 out of a possible 16 cases (randomly selected and ordered) addressing various behavioral targets (eg, smoking, physical activity, diet, and medication adherence). Individual and global competency scores were calculated automatically for the 11 competency items across the 4 cases, providing automatic scores out of 100. From the factorial analysis of variance for the difference in competency and ranking scores, no significant differences were identified between the different case versions across individual and global competency (*P*=.26 to *P*=.97) and ranking scores (*P*=.24 to *P*=.89). The initial tests of internal consistency for rank order among the 24 student participants were in the *acceptable* range (α=.78).

**Conclusions:**

The results suggest that MC-CAT is an internally valid tool to facilitate the evaluation of MC competencies among HCPs and is ready to undergo comprehensive psychometric property analyses with a national sample of health care providers. Once psychometric property assessments have been completed, this tool is expected to facilitate the assessment of MC skills among HCPs, skills that will better support patients in adopting healthier lifestyles, which will significantly reduce the personal, social, and economic burdens of noncommunicable chronic diseases.

## Introduction

The World Health Organization estimates that >71% of deaths worldwide result from noncommunicable diseases (NCDs), including cardiovascular disease, cancer, chronic lung disease, diabetes, and obesity [1]. Despite advances in genetic, pharmacological, and surgical medicine, the prevalence and associated social and economic burden of NCDs are increasing rather than decreasing [2]. This is unsurprising, given that the underlying cause of most NCDs is not biological factors but harmful human behaviors (eg, smoking, physical inactivity, and poor diet) that are poorly addressed by current biomedical approaches [3,4].

As part of offering comprehensive care, health care providers (HCPs) are often responsible for providing some form of behavior change counseling (BCC) to patients who exhibit health risk behaviors. At present, this typically takes the form of offering *persuasive information and advice* [5-7], which has been shown to be either ineffective or counterproductive because patients feel as if they are being *told what to do* [8,9]. When evidence-based BCC approaches are offered by HCPs, they tend to have positive impacts on patient engagement in and the adoption of healthy lifestyle choices [10]. However, one of the most popular of these approaches (ie, motivational interviewing) has generally demonstrated poor uptake by physicians. This has been attributed to perceptions of it being too rigid, taking too much time to implement in practice, and lying outside the physician’s scope of practice [11,12].

To address the limitations of motivational interviewing, we codeveloped (with behavior change experts and HCPs) a new BCC approach called motivational communication (MC), which is based on motivational interviewing and theoretical models of behavior change (eg, self-determination theory [13], social-cognitive theory [14], and transtheoretical model [15]) and incorporates more cognitive behavioral therapy–based components and practical considerations regarding real-world clinical encounters in an NCD management context. Designed as a behavior change communication style specifically developed for HCPs, it is evidence–based and time–efficient and can be used to promote patient engagement, adoption of healthy behaviors, and sustained self-management of chronic conditions [16]. MC was defined as reflecting 11 core communication competencies that have a solid evidence base for behavior change in the context of NCD management [16]. These 11 competencies were summarized under the mnemonic “LEARN tHE BASICs.” These competencies are reflective listening, expressing empathy, demonstrating acceptance, tolerance, and respect, responding to resistance, (not) negatively judging or blaming, (not) expressing hostility or impatience, eliciting *change-talk or* evocation, (not) being argumentative or confrontational, setting goals, providing information neutrally, and being collaborative [16].

After defining MC as well as developing the content of the MC training program to be delivered to HCPs (the *MOTIVATOR* program), we also developed an accompanying MC competency assessment tool to evaluate skill acquisition among HCPs receiving training in this approach. A recent review of the literature on the quality of existing communication assessment tools among HCPs revealed a great deal of heterogeneity over the 45 different assessment tools that were identified. This review also indicated that few tools were developed using appropriate theoretical models (49%), and many failed to clearly define or describe the communication competencies they were designed to evaluate (19%) [17]. In addition, 65% used scoring methods that required extensive training on the part of external assessors, and 93% of the tools required the use of standardized (ie, a person playing the role of a patient; 61%) or real patients (32%) to complete their evaluations [17], potentially undermining the feasibility of implementing this type of evaluation in *real life*. Existing competency assessment tools are hence complex, invasive, time-consuming, and impractical for use in many medical contexts.

Effective, feasible, and user-friendly competency evaluation tools are important not only for assessing the quality and efficacy of training programs but also for ensuring that patients benefit from the BCC methods used by HCPs. Using an integrated knowledge translation (iKT) approach, which is a collaborative model of knowledge production between stakeholders and researchers [18,19], the objective of this study was to develop a new web-based MC competency assessment tool called the Motivational Communication Competency Assessment Test (MC-CAT), to conduct initial internal validity assessments, and to evaluate the ranking and competency score consistency between the base cases and the modified cases as part of a larger iterative development process for this new tool.

## Methods

### Concept

**Figure 1:**
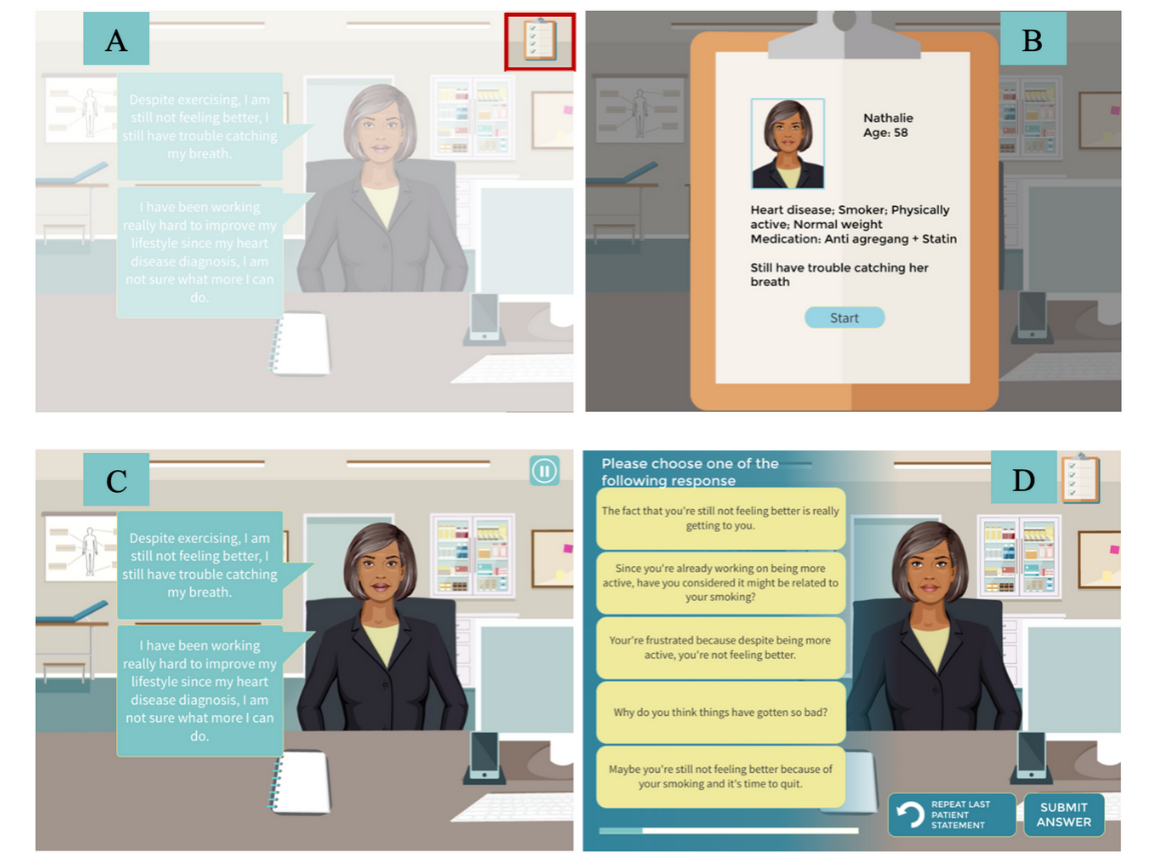
Example of patient chart and interaction between the physician and the virtual patient case. (A) and (B) patient information; always accessible by clicking on the icon in the top right-hand corner; (C) patient’s initial statement (with audio); (D) list of answers; each answer was associated with a score on different motivational communication competencies.

The concept of the web-based MC-CAT assessment tool is to present HCPs with a series of patient cases with a specific behavioral target (eg, engaging in more physical activity). Each case comprises a simulated interaction between a virtual patient and the provider, which focuses on engaging the patient in a discussion about changing their health behavior. Patient information (ie, patient’s picture, age, sex, health condition, health behavior status, and medications) is accessible by clicking on the icon in the top right-hand corner (Figure 1). Each MC-CAT assessment requires completing 4 cases selected at random from a 16-case bank for a total assessment time ranging from 15 to 20 minutes (approximately 5 minutes per case). The cases were designed to be relatively short to maximize the tool’s acceptability and uptake by the busy HCPs.

### Ethics Approval

Ethics approval was provided by the *Centre Intégré Universitaires de Santé et de Service Sociaux du Nord-de-l’île-de-Montréal* (number 2016-1206), and all participants provided informed consent electronically.

### Development

#### Overview

**Figure 2:**
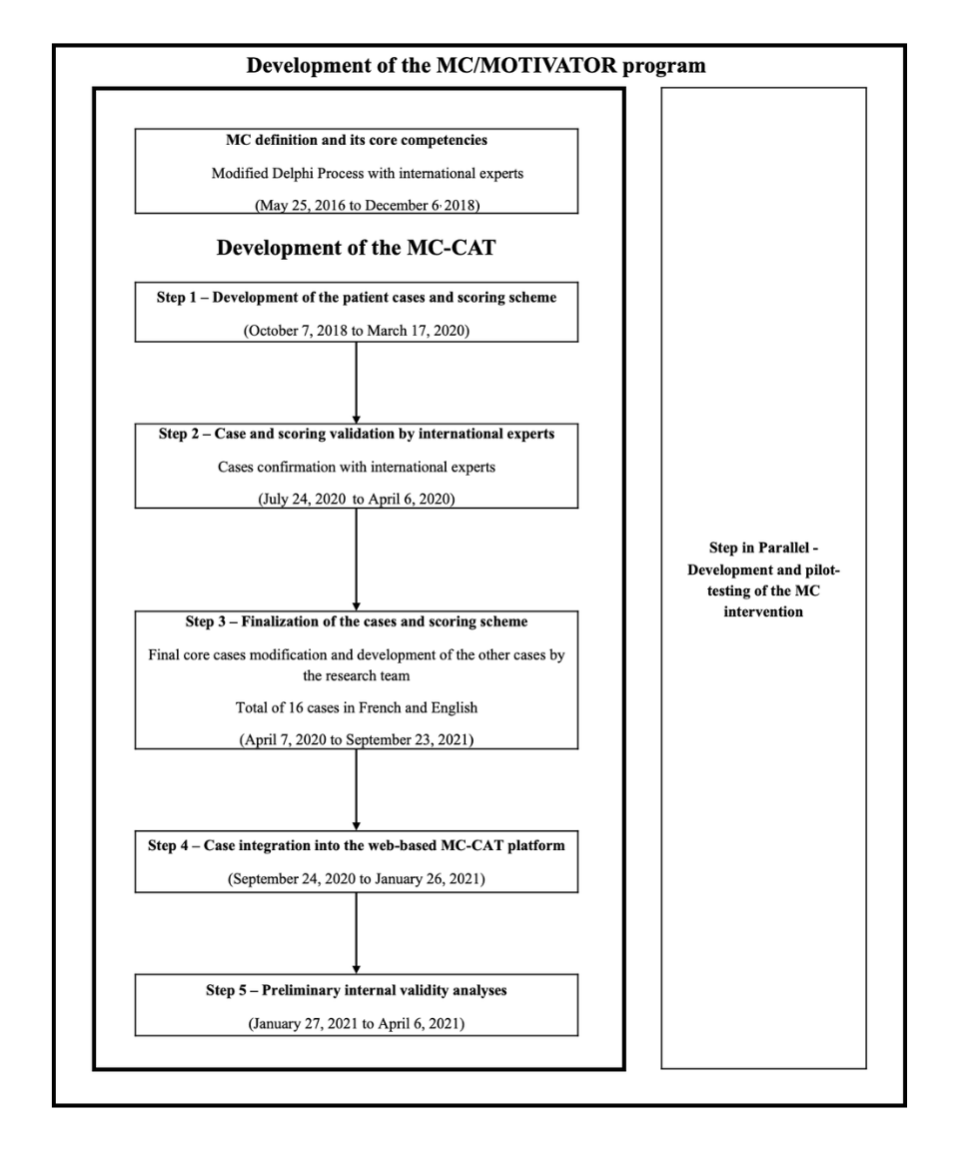
Overview of the MC-CAT development steps. MC: motivational communication; MC-CAT: Motivational Communication Competency Assessment Test.

We have previously defined the 11 core communication competencies of MC [16], which represent the individual competencies evaluated by the MC-CAT. To develop the MC-CAT, we followed a 5-step, mixed methods, iKT approach based on established methods for instrument development and validation, which engaged relevant knowledge users (physicians, HCPs, researchers, and health care administrators) [20-22]. The steps are shown in Figure 2, and are as follows: (1) developing a series of 4 base cases and a scoring scheme to assess the 11 communication competencies of MC, (2) validating the content of the base cases and scoring scheme with international experts, (3) creating 3 alternative versions of the 4 base cases (resulting in a bank of 16 cases, 4 of each type of base case) and translating the cases into French (necessary for a Canadian audience), (4) integrating the cases into the web-based MC-CAT platform, and (5) conducting initial internal validity assessments with a sample of 31 university-allied health students.

#### Step 1: Development of the Patient Cases and Scoring Scheme

The goal of this step was to create 4 base cases (A1, B1, C1, and D1) to assess all 11 MC competencies through simulated medical consultations. One of the primary aims was to develop an assessment tool that would accurately reflect real-life medical consultations targeting health behavior changes in the context of NCD management. To ensure that the behavioral targets of our cases were relevant to clinical practice, we sent an open invitation to Canadian physicians from 4 target specialties known to treat a high volume of patients with NCDs (ie, cardiology, respirology, internal medicine, and general practitioners) to complete a brief (10-minute) web-based survey (LimeSurvey GmbH), which was available in English and French. Invitations were emailed to physicians throughout January 2018 through relevant medical associations (eg, Hypertension Canada and Diabetes Canada) and networks (eg, the Canadian Respiratory Research Network). In this survey, physicians were asked to identify what they believed to be the most important health risk behaviors that they encountered with patients in their efforts to prevent or treat NCDs (checklist with the possibility of adding behaviors) and rank them in order of their relative importance (from most important to least important). This resulted in the identification of the 4 most common health risk behaviors encountered in the context of NCD prevention and management, which would form the behavioral targets of the 4 base cases of MC-CAT*.*

The research team then proceeded to develop 4 base cases around the 4 most important health risk behaviors as identified by physicians, which acted as templates to develop alternate case versions to expand the tool to 16 cases (4 different health behaviors—represented by the letters A, B, C, and D—targeted across 4 different patient cases, numbered from 1 to 4). Base cases were designed to simulate a consultation between an HCP and a patient, which focused on engaging the patient in a conversation about changing their health behavior. Between October 2018 and March 2020, the research team worked on developing 4 base case scripts. The goal was to create a conversation flow beginning with the patient providing an opening statement of concern, after which the HCP was prompted to reply by selecting 1 of 5 multiple-choice responses, reflecting the most MC-consistent responses to the least MC-inconsistent responses (scored from 1 to 5). Each base case included 6 to 7 levels of exchange, each providing the HCP the opportunity to demonstrate ≥1 of the 11 core MC competencies. This resulted in a scoring scheme that indicated the extent to which responses were MC consistent or inconsistent on a 5-point scale (2=very MC consistent, 1=somewhat MC consistent, 0=neither MC consistent nor inconsistent, −1=MC inconsistent, and −2=very MC inconsistent). The scoring format was constructed with a range from +2 to −2 to reflect the nonneutrality of responding in an MC-inconsistent manner (which may be counterproductive for behavior change). Care was taken to provide HCPs with multiple opportunities to demonstrate each of the 11 MC competencies across the 4 cases to ensure that >1 interaction could be used to calculate an individual competency score. MC competency scores were calculated automatically by averaging the individual competency scores across the 4 cases, which were then summed and converted to a global score out of 100, reflecting overall MC competency (theoretical range −93.6 to 100).

#### Step 2: Case and Scoring Validation by International Experts

After creating the 4 base cases (A1, B1, C1, and D1), we proceeded to validate the presence of the different MC competencies reflected in each case, as well as their rank order from most to least MC consistent, using a multi-round survey among a sample of international experts (7/14, 50% women with an average of 21 years of experience in BCC, SD 9 years; Table 1 provides a summary of expert characteristics). The first survey was launched in July 2019 and ended in September 2019. The results were used to calculate the consensus score, reflecting the level of agreement between the experts and the original classifications attributed by the research team. The original classifications were considered *good* if the agreement with the experts was perfect, *acceptable* if the expert rank was a +1-point or –1-point deviation from the rank the research team had indicated (eg, ranked 5 instead of 4), and *poor* if the expert order was a +2-point –2-point deviation from the research team rank (eg, ranked 1 instead of 3). These results were used to make minor modifications to some response items based on the criteria summarized in Table 2. This resulted in the construction of a second confirmatory survey, in which the same experts were asked to confirm their agreement with the new competency classification and ranking. The survey was launched in October 2019 and ended in April 2020.

**Table 1:** Demographic information of health care providers and international experts.

Variable				Health care providers (N=80), n (%)		International experts (N=14), n (%)
**Gender**						
	Women			36 (45)		7 (50)
	Men			44 (55)		7 (50)
**Language spoken**						
	English speaking			30 (38)		12 (86)
	French speaking			50 (62)		2 (14)
**Age (years)**						
	<30			2 (3)		—^a^
	30-39			27 (34)		3 (21)
	40-49			9 (11)		6 (43)
	50-59			19 (24)		2 (14)
	60-69			23 (29)		2 (14)
	≥70			—		1 (7)
**Duration of practice (years)**						
	0-5			20 (25)		—
	6-10			9 (11)		1 (7)
	11-15			7 (9)		3 (21)
	16-20			3 (4)		6 (43)
	21-25			13 (16)		—
	26-30			10 (13)		1 (7)
	>30			18 (23)		3 (21)
**Province of practice or country**						
	**Canada**					
		Alberta	2 (3)		2 (14)	
		British Columbia	3 (4)		—	
		New Brunswick	1 (1)		—	
		Nova Scotia	2 (3)		2 (14)	
		Ontario	17 (21)		3 (21)	
		Quebec	55 (69)		4 (29)	
	Sweden			—		1 (7)
	United Kingdom			—		1 (7)
	United States			—		1 (7)

^a^Data not available.

**Table 2:** Criteria for case modification after evaluation by behavior change experts.

Criteria			Modifications
**Ranking the choices of the different base cases physician’s options**			
	≥70% of good agreement^a^ and ≤10% of poor agreement^b^ between external experts and research team rankings^c^	No modification	
	≥90% of good and acceptable^d^ agreement and ≤10% of poor agreement between external experts and research team rankings	No modification	
**Competency identification**			
	≥70% agreement between external experts and research team identification	Competencies kept or added if there was agreement	
	Between 40% and 69% of agreement between external experts and research team identification	Competencies may be kept or added depending on the research team’s consensus	
	≤30% agreement between external experts and research team identification	Competencies deleted	

^a^If agreement with the experts and the research team was perfect.

^b^The expert order was +2 or –2 deviations in rank from the research team (eg, ranked 1 instead of 3).

^c^If this criterion is not met, the modifications must result in a minimum of 70% perfect agreement or 90% perfect and partial agreement and <10% of complete disagreement.

^d^If the expert rank was +1 or –1 point deviation in rank from the research team (eg, ranked 5 instead of 4).

#### Step 3: Finalization of the Cases and Scoring Scheme

##### Overview

One of the aims was to design the MC-CAT to allow for the variation (or flexible *programming*) in the demographic variables of the virtual patients (ie, age, sex, race, culture, and language), NCDs, and contextual variables (eg, personal information) across cases. To achieve this, we adapted the 4 base cases to expand the test bank (ie, create alternate versions of the 4 base cases) without altering the core cases’ original structure (allowing us to maintain the integrity of the scoring algorithm and conversation branching across established at step 2). As such, every MC-CAT assessment of 4 randomly selected cases should maintain the integrity and psychometric properties by including a variant of each of the 4 original cases. This resulted in the creation of 16 unique patient cases (cases A1 to A4, B1 to B4, C1 to C4, and D1 to D4) reflecting a variety of cases that would be appropriate for multiple types of NCD management situations and relevant across different medical specialties. These 16 cases were then formally translated into French (using back translation to ensure equivalence) [23] to permit use with French-speaking physicians, which is relevant in the Canadian context.

##### Scoring Algorithm

The MC-CAT provides a subscale score for each of the 11 MC competencies, as well as a global score summarizing overall MC competency. Individual competency scores were calculated by considering the number of times the person had chosen responses that included a certain competency (eg, reflective listening) across the 4 cases, divided by the total number of times the competency could have been chosen across the evaluation, and multiplied by the relative proportion of opportunities to demonstrate that competency over all the competencies evaluated.

To obtain scores for global competency, the scores for the positive competencies (reflective listening; expressing empathy; demonstrating acceptance, tolerance, and respect; responding to resistance; eliciting *change-talk* or evocation; setting goals; providing information neutrally; and being collaborative) are aggregated together, and the negative competencies (negatively judging or blaming, expressing hostility or impatience, and being argumentative or confrontational) are subtracted from the sum, as reflected in the following equation:

Competencies score (%) = ([reflective listening / number of reflective listening occasions × percentage of reflective listening cases] + [expressing empathy / number of expressing empathy occasions × percentage of expressing empathy cases] + [evocation / number of evocation occasions × percentage of evocation cases] + [responding to resistance / number of responding to resistance occasions × percentage of responding to resistance cases] + [setting goals / number of setting goals occasions × percentage of setting goals cases] + [acceptance, tolerance, and respect / number of acceptance, tolerance, and respect occasions × percentage of acceptance, tolerance, and respect cases] + [being collaborative / number of being collaborative occasions × percentage of being collaborative cases] + [providing information neutrally / number of providing information neutrally occasions × percentage of providing information neutrally cases]) – ([hostility + negatively judging + argumentative] × [1 / number of hostility, negatively judging, argumentative occasion cases × 100]) **(1)**

The score for the ranking (ie, whether the physician selected the most consistent response with MC [1] or the least consistent response with MC [5]) is calculated by adding the number of times a participant selected the ranks of response choices multiplied by a constant ranging from 2 to −2 (eg, 10 times the second choice is multiplied by 1). The following is the equation for ranking scores:

Ranking score (%) = ([number of first choices × 2] + [number of second choices × 1] + [number of third choices × 0] + [number of fourth choices × −1] + [number of fifth choices × −2]) / (50×100) **(2)**

#### Step 4: Case Integration Into the Web-Based MC-CAT Platform

Following the completion of steps 2 to 3, the first web-based computerized version of the MC-CAT (version 1.0), including 32 virtual patient cases (16 in French and 16 in English, which are identical and translated) and a demographic questionnaire (including sex, age, location of practice, primary medicine specialty, clinical setting, years of practice, number of patients, and physicians’ attitudes toward addressing health risk behaviors) was designed and created in collaboration with 42 Comets Inc, a software developer with expertise in the creation of electronic education and training programs. To test the user interface of the MC-CAT, we conducted user experience research with 27 volunteer graduate students, HCPs, and behavior change experts also involved in step 2. The goal was to determine (1) the clarity of the instructions and tasks, (2) the navigability of the platform, (3) the synchronicity between audio and video information, and (4) the acceptability of the duration of the assessment. The responses were used to refine the aspects of the web-based interface to optimize the functionality of the program.

#### Step 5: Preliminary Internal Validity Analyses

##### Overview

We refined the aspects of the web-based interface based on user experience testing. The final step in the development process was to collect preliminary psychometric properties of the MC-CAT from a sample of MC-naïve undergraduate allied HCP students who were recruited via email and invited to complete 2 MC-CAT assessments approximately 12 weeks apart. Each assessment involved completing a basic sociodemographic questionnaire followed by the MC-CAT (4 randomly selected cases from the 16-case bank, 1 from each series A to D).

##### Case Consistency Analyses

To evaluate the consistency of the ranking and competency scores between the base cases (A1, B1, C1, and D1) and the modified cases (cases A2 to A4, B2 to B4, C2 to C4, and D2 to D4), a factorial ANOVA for the difference in competency and ranking scores was used to determine differences between case variations (ie, case A1 vs A2 vs A3 vs A4) across individual competency scores, global competency scores, and ranking scores.

##### Internal Consistency of the Tool

The internal consistency of the MC-CAT was obtained by calculating the Cronbach α coefficient [24]. The rank of the response selected by the participant (ranging from 1=most consistent with MC to 5=least consistent with MC) for each of the response choices per case (A, B, C, and D) and for the entire MC-CAT assessment (all responses over 4 cases) were used to calculate the coefficient. Thus, this analysis aimed to determine whether the responses chosen by the participants were consistent across the 4 cases. An acceptable score for the Cronbach α coefficient is between .70 and .95 [24,25], which was adopted as our target criterion for moving forward with the tool.

## Results

### Step 1: Development of the Patient Cases and Scoring Scheme

We received 154 surveys, of which 80 (52%) physicians had complete data (n=22, 28% cardiologists; n=22, 28% respirologists; n=15, 19% internists; and n=21, 26% general practitioners) and were included in the analyses. The mean age was 49 (SD 12.9) years. Of the 80 physicians, 44 (55%) were male, and 50 (63%) identified French as the first language. The mean duration of practice of the physicians was 18 (SD 11.9) years, and 69% (55/80) of physicians were working in a university hospital setting and had a mean of 38 (SD 24.2) weekly NCD consultations (Table 1 presents the participants’ information).

The health risk behaviors most frequently identified by physicians were physical inactivity (75/80, 94%), smoking (73/80, 91%), medication nonadherence (71/80, 89%), and unhealthy diet (69/80, 86%). Physicians ranked smoking first, medication nonadherence second, physical inactivity third, and unhealthy diet fourth in the list of most important health risk behaviors to address in the context of NCD management. The following health risk behaviors were perceived as the most prevalent among their patients: (1) physical inactivity (mean 58%, SD 19.7%), (2) unhealthy diet (mean 47.2%, SD 18.2%), and (3) difficulty in managing stress (mean 44.8%, SD 18.9%). On the basis of these results, we designed 4 core cases of the MC-CAT to feature cases with health risk behaviors—smoking, physical inactivity, nonadherence to medication, and poor diet—representing a range of NCDs (eg, obesity, asthma, diabetes, and hypertension) [26]. The average number of opportunities was 11.4 (SD 6.4, range 2-24).

### Step 2: Case and Scoring Validation by International Experts—Content Validity

The initial percentage of agreement between our classification and the experts’ for the rank order of responses across all 4 base cases was 60.9% (SD 14.0%; range 37.1%-84.3%). The competency identification agreement across all 4 base cases was 44.9% (SD 8.4%; range 30.5%-60.2%). In response to these results and considering the specific feedback provided by our international experts (74 comments over the 4 cases), we made 8 modifications to the rank ordering of statements and 23 modifications to aspects of the dialog (eg, making a statement more or less consistent with MC; Table 3). The experts were then asked to assess whether they agreed with the new rankings of the modified cases and the competencies identified. After this evaluation, an increase was noted in agreement for both the rank order (mean 87.6%, SD 16.8%; range 16.7%-100%) and the competency identification (mean 78.1%, SD 14.3%; range 48.6%-100%), which is considered as an acceptable level of agreement [27].

**Table 3:** Percentage agreement of the rank order of responses across all 4 base cases.

Choice of response for each case			Agreement							Agreement after modification				
			Good		Acceptable		Poor		Good			Acceptable		Poor
**Case A, (%)**														
	1	57.1		25.7		17.1		78.3			12.9		10	
	2	58.6		37.1		4.3		70			25.7		4.3	
	3	37.1		38.6		24.3		73.3			26.7		0	
	4	70		27.1		2.9		80			20		0	
	5	60		30		10		86.7			13.3		0	
	6	45.7		40		14.3		70			28.3		1.7	
	Mean (SD)	54.8 (11.6)		33.1 (6.2)		12.2 (8.1)		76.4 (6.5)			21.1 (6.8)		2.7 (4.0)	
**Case B, (%)**														
	1	47.1		27.1		25.7		80			15		3.3	
	2	57.1		27.1		15.7		91.7			6.7		1.7	
	3	78.6		18.6		2.9		78.3			20		0	
	4	65.7		30		4.3		85			11.7		1.7	
	5	45.7		48.6		5.7		86.7			13.3		0	
	6	68.6		28.6		2.9		81.7			16.7		1.7	
	Mean (SD)	60.5 (12.9)		30 (9.9)		9.5 (9.3)		83.9 (4.9)			13.9 (4.5)		1.4 (1.2)	
**Case C, (%)**														
	1	78.6		15.7		4.3		81.7			13.3		3.3	
	2	45.7		44.3		10		86.7			6.7		6.7	
	3	47.1		40		12.9		80			16.7		3.3	
	4	74.3		22.9		2.9		70			26.7		3.3	
	5	78.6		17.1		4.3		81.7			13.3		5	
	6	84.3		14.3		1.4		86.7			13.3		0	
	7	57.1		31.4		11.4		80			20		0	
	Mean (SD)	66.5 (16.2)		26.5 (12.2)		6.7 (4.6)		81.0 (5.6)			15.7 (6.3)		3.1 (2.4)	
**Case D, (%)**														
	1	73.3		18.3		8.3		73.3			18.3		8.3	
	2	43.3		53.3		3.3		90			10		0	
	3	76.7		20		3.3		76.7			20		3.3	
	4	73.3		23.3		3.3		93.3			6.7		0	
	5	60		26.7		13.3		93.3			6.7		0	
	6	48.3		40		8.3		81.6			16.7		1.7	
	Mean (SD)	62.5 (14.2)		30.3 (13.7)		6.6 (4.1)		84.7 (8.7)			13.1 (6.0)		2.2 (3.3)	
Overall agreement, mean (SD)			61.2 (13.8)		29.8 (10.5)		8.7 (6.8)		81.5 (6.9)			15.8 (6.4)		2.5 (2.8)

### Steps 3 and 4: Finalization of the Cases, Scoring Scheme, and Integration Into the Web-Based MC-CAT Platform

As part of the log-in process, HCPs were asked to enter basic demographic information, including language preference, age, sex, and specialty, the latter of which was used to present HCPs with cases in their area of practice. Each case began by presenting respondents with relevant patient information (age, sex, diagnosis, and basic clinical information) in a *file* located at the top right-hand corner of the screen and accessible anytime during the assessment (Figure 2). The physician was then informed of the behavioral target (eg, increasing physical activity) and instructed to engage the patient in a conversation about changing their behavior. The case always started with a patient expressing ambivalence about health behavior changes. The physician was then directed to select a response from 1 of 5 randomly ordered options (Figure 1 provides a visual example of a case). Each response corresponded to an opportunity to demonstrate ≥1 of the 11 core MC competencies, which are assessed multiple times per case and across cases and averaged to obtain a score for that competency (Table 4 presents the distribution of the competencies for each base case).

Of the 27 responses received during user experience testing, 10 (37%) comments reflected audio and visual elements (eg, synchronization of the voice with the appearance of the text and the mouth movement of the virtual patient), and 19 (70%) comments reflected instruction elements (eg, lack of clarity in sections of the consent form; typos). On the basis of this, the 29 comments were addressed by the research team and 42 Comets Inc when creating a new version of the MC-CAT platform (version 2.0).

**Table 4:** Distribution of competencies for each of the 4 core cases.

MC-CAT^a^ competency	Possibility of expressing target behavior						Example
	Case A: physical activity, n (%)	Case B: smoking cessation, n (%)	Case C: healthy diet, n (%)	Case D: medication adherence, n (%)	Total, N		
Reflective listening	3 (19)	4 (25)	5 (31)	4 (25)	16	“So you recognize the potential benefits of a healthier diet, but it's challenging given your line of work.”	
Expressing empathy	2 (20)	3 (30)	3 (30)	2 (20)	10	“Changing your daily eating habits when there are barriers can be challenging. But exploring the benefits may help.”	
Eliciting “change-talk” or evocation	2 (22)	2 (22)	3 (33)	2 (22)	9	“You said you were fed up with feeling breathless, and recognize that smoking might be the cause. What would increase your confidence in your ability to quit?”	
Responding to resistance	2 (33)	2 (33)	0 (0)	2 (33)	6	“It might help to know the benefits of exercise. Tell me what you think you would be able to do if you were in better shape?”	
Goal setting	2 (29)	1 (14)	2 (29)	2 (29)	7	“Cooking would be a great place to start! And if it's something you enjoy, you are more likely to stick with it. What is your plan to get started?”	
Demonstrating acceptance, tolerance, and respect	4 (31)	2 (15)	4 (31)	3 (23)	13	“It sounds like a great plan, and your willingness to getting more information this weekend demonstrates how important this is to you.”	
Being collaborative	2 (25)	2 (25)	1 (13)	3 (38)	8	“It sounds like we just need to find a routine that works for you. Could we explore some options together?”	
(Not) expressing hostility or impatience	1 (9)	4 (36)	5 (45)	1 (9)	11	“If you want to avoid exacerbating your diabetes, you need to commit to a diet change, sooner rather than later.”	
(Not) negatively judging or blaming	5 (25)	6 (30)	4 (20)	5 (25)	20	“I think that's a good place to start, all you need to do is follow through.”	
(Not) being argumentative or confrontational	6 (25)	6 (25)	6 (25)	6 (25)	24	“Yes, but since you lack confidence you should also get behavioral counselling, you don’t want to fail again!”	
Providing information neutrally	0 (0)	1 (50)	0 (0)	1 (50)	2	“There are several options: nicotine replacement therapy, medications, and behavioral counselling have all been shown to be effective. What do you think would work best for you?”	
Total	29 (23)	33 (26)	33 (26)	31 (25)	126	—^b^	
Exchanges	6 (24)	6 (24)	7 (28)	6 (24)	25	—	

^a^MC-CAT: Motivational Communication Competency Assessment Test.

^b^Data not available.

### Step 5: Preliminary Internal Validity Analyses

We received 24 MC-CAT responses from undergraduate allied HCP students. All participants had completed both assessments (17/24, 71% female; living in the province of Quebec, Canada; English speaking; with 0-5 years of practice in BCC and no previous training in MC).

#### Case Consistency Analyses

**Figure 3:**
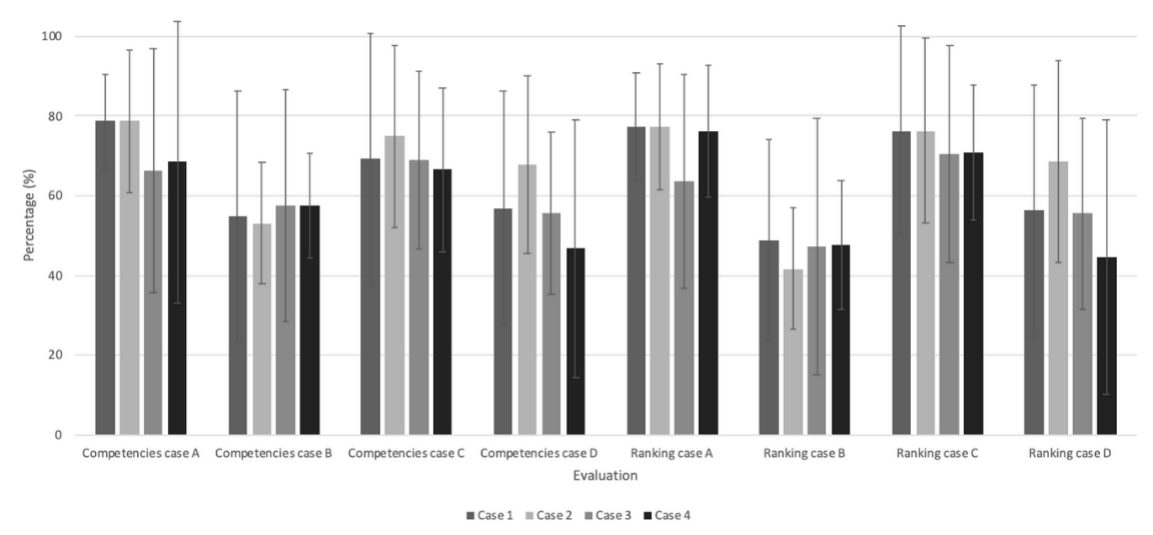
Score differences between each case version for time 1 (N=24).

**Figure 4:**
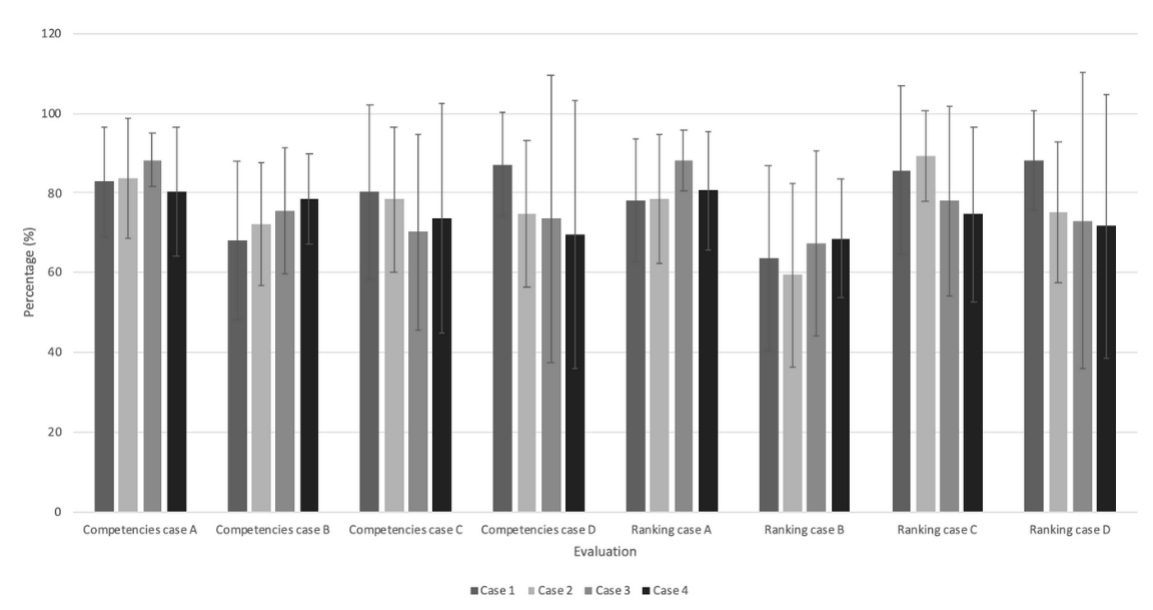
Score differences between each case version for time 2 (N=24).

To identify possible differences between the different versions of the base cases (cases A1 to A4, B1 to B4, C1 to C4, and D1 to D4), ANOVAs were performed by comparing the competency and ranking scores. No significant differences were identified between the different versions of the case across the 2 measurement times (Figures 3 and 4; Table 5).

**Table 5:** Global competency and ranking weighted scores at assessments 1 and 2 (N=24).

Cases				All (%), mean (SD)		Case 1 (%), mean (SD)		Case 2 (%), mean (SD)		Case 3 (%), mean (SD)		Case 4 (%), mean (SD)		Difference between cases, *P* value
**Precourse**														
	**Competency (global competency score** **64.9, SD 19.0)**													
		Case A	73.1 (25.6)		78.7 (11.6)		78.7 (18.0)		66.3 (30.6)		68.5 (35.4)		.51	
		Case B	55.8 (24.2)		54.9 (31.4)		53.1 (15.3)		57.5 (29.1)		57.5 (13.1)		.97	
		Case C	70.0 (32.3)		69.4 (31.5)		75.0 (22.8)		68.9 (22.1)		66.6 (20.6)		.82	
		Case D	56.8 (27.4)		56.9 (29.2)		67.9 (22.2)		55.5 (20.4)		46.8 (32.4)		.26	
	**Ranking (global ranking score** **65.8, SD 17.4)**													
		Case A	73.6 (18.9)		77.3 (13.5)		77.4 (15.8)		63.6 (26.9)		76.3 (16.6)		.24	
		Case B	46.4 (23.2)		49.0 (25.2)		41.7 (15.2)		47.2 (32.0)		47.7 (16.3)		.89	
		Case C	73.5 (22.6)		76.4 (26.1)		76.3 (23.3)		70.4 (27.2)		70.9 (16.9)		.87	
		Case D	56.3 (30.1)		56.2 (31.6)		68.6 (25.3)		55.6 (23.9)		44.6 (34.4)		.24	
**Postcourse**														
	**Competency (global competency score** **77.6, SD 16.5;*P*<.001)**													
		Case A	83.7 (13.3)		82.8 (13.7)		83.6 (15.0)		88.2 (6.6)		80.4 (16.1)		.54	
		Case B	73.3 (15.9)		68.2 (19.8)		72.0 (15.4)		75.5 (15.8)		78.4 (11.4)		.50	
		Case C	75.3 (23.5)		80.1 (21.8)		78.3 (18.2)		70.2 (24.5)		73.5 (28.7)		.68	
		Case D	77.7 (26.5)		87.2 (12.8)		74.6 (18.4)		73.5 (36.2)		69.6 (33.6)		.32	
	**Ranking (global ranking score** **77.8, SD 16.6;*P*<.001)**													
		Case A	81.3 (14.1)		78.2 (15.4)		78.5 (16.1)		88.2 (7.5)		80.6 (14.8)		.26	
		Case B	64.3 (21.6)		63.6 (23.4)		59.4 (23.1)		67.3 (23.2)		68.5 (14.9)		.72	
		Case C	81.5 (21.1)		85.7 (21.1)		89.3 (11.5)		78.1 (23.8)		74.7 (22.0)		.37	
		Case D	78.4 (26.5)		88.2 (12.5)		75.0 (17.7)		73.1 (37.0)		71.7 (32.9)		.31	

#### Internal Consistency of the Tool

The MC-CAT tool showed acceptable values of internal consistency for global scores (25 items) at both time 1 (α=.78) and time 2 (α=.80).

## Discussion

### Principal Findings

The objectives of this study were to develop the MC-CAT, a new, web-based, *user-friendly* tool for assessing communication competencies among HCPs in the context of changing health behaviors among patients with NCDs, conduct an initial internal validity assessment, and evaluate score consistency between the base and modified cases. The MC-CAT was designed to simulate clinical interactions with *virtual* patients to provide both global and specific scores for 11 core communication skills [16]. It was co-designed in collaboration with key stakeholders (ie, physicians, HCPs, researchers, and health care administrators) using an iKT approach to ensure its clinical relevance and feasibility for use in practice. The web-based platform was also user-tested to ensure ease of navigability among target users.

The results of this 5-step mixed methods study indicate that the MC-CAT demonstrates acceptable levels of internal consistency for the global competency score (α=.78-.80), and little variance was found across different versions of the 4 base cases. This level of internal consistency is higher than the levels observed in many existing communication assessment tools such as the Pediatric Consultation Assessment Tool and the Four Habits Coding Scheme, which had Cronbach α values between .52 [28] and .66 [29]. However, it was slightly lower than the levels seen in other tools (eg, the Council of Emergency Medicine Residency Directors Standardized Direct Observation Assessment Tool [30] or the Doctors’ Observable Use of Self-Efficacy Enhancing Interviewing Techniques measure [31], with Cronbach α=.93 and .94, respectively). Several factors can affect the results of a tool’s internal consistency analysis (also considered a measure of scale reliability), such as the number of participants included in the analysis (varying between 19 and 82 participants for these 4 tools, in contrast to 32 participants for our analyses), the potential for evaluation biases associated with assessment methods (eg, self-report surveys and observational scales vs an objective scoring algorithm), and the number of items (varying between 10 and 26 items for these 4 tools). However, using a rigorous development process, we have developed an assessment tool that met our internal validity criterion (ie, α between .70 and .95), and we are satisfied that we can move forward with internal and external validation among HCPs.

### Comparison With Prior Work

To our knowledge, this is the first study to describe the development of a web-based interactive communication skills assessment tool for HCPs [17]. The MC-CAT tool was also developed in parallel with a theory-driven and evidence-informed MC training framework for HCPs [16]. As such, the MC-CAT addresses the shortcomings of approximately 50% of the 45 existing communication competency assessment tools [17], which our recent review revealed were not informed by established theories of communication or behavior change. Furthermore, most tools did not explicitly define the communication competencies they were designed to assess [17], unlike MC-CAT, which was specifically designed to assess the 11 core communication competencies of MC, which HCPs and behavior change experts identified as being the most critical for changing health behaviors in the context of NCD management [16]. We developed a tool to assess MC competencies [16] as MC has become an increasingly popular communication style among HCPs [32,33]. The fact that MC-CAT assesses all MC competencies and not a subset of these skills also overcomes the limitations of previous tools that have not been developed to provide comprehensive assessments of specific communication frameworks [17]. The total scores on the MC-CAT also reflected the relative importance of each individual competency proportional to its use in practice. In other words, communication skills that are used more frequently during patient consultations (eg, asking open *evocative* questions, reflective listening, and expressing empathy) are given greater weight in the final scoring. This is an important strength of the tool, as we are aware of no existing tools that take into account the *real-world* frequency with which certain skills are used in their scoring algorithms. We also ensured that the different versions of our 4 base cases (cases A1 to A4, B1 to B4, C1 to C4, and D1 to D4) were comparable in terms of competency and ranking scores. This means that our adaptations were consistent with the original scoring algorithm and that we can use them to create further adaptations of the 4 base cases to further extend the case bank of the tool.

One of the most useful and attractive features of the MC-CAT is that it is scored automatically based on a preprogrammed algorithm, which eliminates the need for external raters to conduct assessments (ie, interrater reliabilities). The need for external, trained raters is a feature of all existing assessment tools [17]. Although more rigorous than self-reported assessments, a manual rating is associated with significant costs in terms of time and complexity. The fact that the MC-CAT is scored automatically also reduces the potential biases associated with the subjective nature of rater assessments and eliminates the need for multiple raters to assess agreement, which removes time and complexity. Indeed, in previous studies using external raters to assess physician communication competencies, training time averaged 14 hours and ranged from 1.5 to >90 hours [17], which may not be feasible to implement in practice. Most previous studies (61%) also failed to standardize the training of external raters [17], which can greatly affect the fidelity of the coding process and does not allow for the comparison of one evaluation with another. Finally, the MC-CAT was specifically designed to address the practical constraints of many NCD-focused physicians who may not have the time to undergo complex evaluations [11]. The MC-CAT tool is completed on the web using any electronic device and takes between 15 and 20 minutes to complete, which are features that our HCP collaborators have indicated as both acceptable and feasible.

### Study Limitations and Strengths

First, this study may be limited by the fact that we did not specifically include patients with NCDs as part of our stakeholder groups. The rationale was that our target users were HCPs; hence, our focus was on engaging various physicians, HCPs, and health administrator stakeholders. There is already an evidence base demonstrating that patients whose physicians use MC-type approaches feel more understood, have more trust in their providers, are more adherent to treatment, are more satisfied with their care, and have better outcomes [10,34-38]. As such, our goal was not to validate this work but rather to focus on how to facilitate the implementation of these approaches into practice. Second, although the MC-CAT includes a range of cases that are intended to reflect real-world clinical encounters, it was not possible to create cases that reflected all behavioral issues involved in these diseases, which may limit the generalizability of the tool. Similarly, we attempted to include cases that reflected patient diversity in terms of sociodemographic characteristics (ie, age, sex, and race or culture); however, it was not possible to include all combinations and permutations of these characteristics, which may be seen as a limitation. However, now that we have validated the scoring integrity of our 4 base cases and their alternate versions, our next step is to create additional adaptations that will increase the heterogeneity of our case bank. Finally, the MC-CAT relies on computer and internet access, which may not be readily available to some providers.

Despite these limitations, this study also has several notable strengths. Critical to successfully developing a valid and reliable assessment tool, we integrated several key stakeholder groups in all steps of the development process (eg, content, testing, and recruitment) using the knowledge transfer cycle as the basis of our iKT strategy [18]. This is expected to optimize the uptake and impact of assessment tools in clinical practice and research. In addition, we did not neglect the design of the web-based platform and conducted careful user-testing using the User Experience Framework [39] to assess appeal, clarity, and navigability. This framework provides several dimensions to consider when designing and testing a web-based tool, presented on a continuum from abstract to concrete regarding visual design, interaction design, and functional specifications. Through this process, we further refined the tool to make it more *user-friendly* and intuitive. Another strength of our development process is that we created multiple versions of our base cases and tested whether they were comparable using a factorial analysis, which revealed no significant differences between cases on competency and ranking scores. This increases our confidence that the MC-CAT is now ready for comprehensive psychometric property analyses among target users (ie, HCPs: nurses and physicians), which is the next step in the development process.

### Conclusions

The MC-CAT is a new web-based, interactive, user-friendly MC assessment tool that was codeveloped with a range of relevant stakeholders. The results demonstrated acceptable internal consistency for global competency scores, which indicates that it is ready for more comprehensive psychometric property analyses, including both internal and external validity tests (eg, positive and negative predictive values and convergent validity) in a national sample of HCPs across disciplines. We will continue to use an iterative approach during subsequent phases of development, and we are prepared to further refine the tool and its scoring algorithm as needed. Once developed, the MC-CAT will be the first web-based MC assessment tool that can be easily and widely accessed by a variety of HCPs and can be used not only as an evaluation tool but also as an adjunct to the MC training programs. Its accessibility, convenience, and *user-friendliness* are expected to increase the uptake and improve the quality of MC training programs designed to improve HCPs’ ability to effectively motivate and support patients to adopt healthy behaviors in the context of NCD prevention and management.

## Abbreviations

BCC: behavior change counseling
HCP: health care provider
iKT: integrated knowledge translation
MC: motivational communication
MC-CAT: Motivational Communication Competency Assessment Test
NCD: noncommunicable disease
